# AMPK signaling inhibits the differentiation of myofibroblasts: impact on age-related tissue fibrosis and degeneration

**DOI:** 10.1007/s10522-023-10072-9

**Published:** 2023-11-02

**Authors:** Antero Salminen

**Affiliations:** Department of Neurology, Institute of Clinical Medicine, University of Eastern Finland, P.O. Box 1627, 70211 Kuopio, Finland

**Keywords:** Ageing, Fibroaging, Immunosuppression, Lifespan, Metformin, Rapamycin, ROS

## Abstract

Disruption of the extracellular matrix (ECM) and an accumulation of fibrotic lesions within tissues are two of the distinctive hallmarks of the aging process. Tissue fibroblasts are mesenchymal cells which display an impressive plasticity in the regulation of ECM integrity and thus on tissue homeostasis. Single-cell transcriptome studies have revealed that tissue fibroblasts exhibit a remarkable heterogeneity with aging and in age-related diseases. Excessive stress and inflammatory insults induce the differentiation of fibroblasts into myofibroblasts which are fusiform contractile cells and abundantly secrete the components of the ECM and proteolytic enzymes as well as many inflammatory mediators. Detrimental stresses can also induce the transdifferentiation of certain mesenchymal and myeloid cells into myofibroblasts. Interestingly, many age-related stresses, such as oxidative and endoplasmic reticulum stresses, ECM stiffness, inflammatory mediators, telomere shortening, and several alarmins from damaged cells are potent inducers of myofibroblast differentiation. Intriguingly, there is convincing evidence that the signaling pathways stimulated by the AMP-activated protein kinase (AMPK) are potent inhibitors of myofibroblast differentiation and accordingly AMPK signaling reduces fibrotic lesions within tissues, e.g., in age-related cardiac and pulmonary fibrosis. AMPK signaling is not only an important regulator of energy metabolism but it is also able to control cell fate determination and many functions of the immune system. It is known that AMPK signaling can delay the aging process via an integrated signaling network. AMPK signaling inhibits myofibroblast differentiation, e.g., by suppressing signaling through the TGF-β, NF-κB, STAT3, and YAP/TAZ pathways. It seems that AMPK signaling can alleviate age-related tissue fibrosis and degeneration by inhibiting the differentiation of myofibroblasts.

## Introduction

Tissue fibroblasts possess a remarkable phenotype plasticity which is a beneficial property when they strive to maintain structural integrity and tissue homeostasis (LeBleu and Neilson 2020; Plikus et al. 2021; Salminen 2023a). Fibroblasts secrete the major proteins in the extracellular matrix (ECM), such as collagens, fibronectin, elastins, and proteoglycans and accordingly, they are able to remodel the ECM by producing the proteolytic enzymes which degrade its structural components. In addition, fibroblasts can act as immune regulators, i.e., they are able to produce inflammatory mediators and display both pro-inflammatory and anti-inflammatory phenotypes as a response to changes in the tissue microenvironment (Mhaidly and Mechta-Grigoriou 2020; Davidson et al. 2021; Schuster et al. 2021). In pathological states, tissue-resident fibroblasts can differentiate into myofibroblasts which are able to display both fibrogenic and immunosuppressive properties. Fibroblasts also co-operate with immune cells, i.e., fibroblasts can recruit immune cells into injured tissues and moreover, immune cells, such as monocytes and macrophages, can become transdifferentiated into myofibroblasts (Meng et al. 2016; Vierhout et al. 2021). Fibroblasts can also be switched into a state of cellular senescence and in that situation these pro-inflammatory cells can promote the accumulation of fibrotic lesions and induce degenerative processes (Tigges et al. 2014; Hernandez-Gonzalez et al. 2021). Fibroblasts, especially myofibroblasts and senescent fibroblasts, have a crucial role in many pathological conditions, such as cancers, tissue injuries, and many fibrotic and inflammatory states. Interestingly, the structural alterations of the ECM involving fibrotic lesions are hallmarks of the aging process (Freitas-Rodríguez et al. 2017; Selman and Pardo 2021). Currently, it is known that several enhancers of the aging process, such as oxidative stress and inflammatory mediators, are potent inducers of myofibroblast differentiation and the deposition of fibrotic lesions, whereas anti-aging treatments, such as exposure to either metformin or rapamycin, have been shown to be able to inhibit the differentiation of myofibroblasts and suppress fibrogenesis (see below).

The AMP-activated protein kinase (AMPK) is not only an important regulator of energy metabolism but it is also involved in several other functions, e.g., cell fate determination as well as innate and adaptive immunity (Hardie et al. 2012; Young et al. 2016; Hardie and Lin 2017; Ma et al. 2017; Salminen et al. 2019). AMPK signaling targets a mounting number of substrates, many of which are signaling proteins controlling diverse cellular activities. Interestingly, it is known that AMPK signaling is able to modulate many of the functions implicated in the aging process, e.g., (1) the levels of oxidative and endoplasmic reticulum stresses (Kim et al. 2015; Rabinovitch et al. 2017), (2) the activity of autophagy (Kim et al. 2011; Ge et al. 2022), (3) the appearance of cellular senescence (Han et al. 2016), (4) the accumulation of ECM components (Luo et al. 2015), and (5) chronic low-grade inflammation (Salminen et al. 2011). Currently, AMPK signaling has been recognized as an important anti-aging regulator which also has therapeutic benefits in many age-related diseases (Salminen and Kaarniranta 2012; Burkewitz et al. 2014; Li et al. 2020). Interestingly, there is abundant evidence that AMPK signaling inhibits the differentiation of myofibroblasts which are involved not only in the regulation of integrity of the ECM but they also co-operate with immune cells in pathological conditions (see below). I will first describe the properties of myofibroblasts and then examine the AMPK-dependent signaling mechanisms which inhibit myofibroblast differentiation and thus are able to attenuate the appearance of fibrotic lesions in both the aging process and age-related diseases. It seems plausible that AMPK signaling can alleviate age-related tissue degeneration by inhibiting myofibroblast differentiation and the deposition of fibrotic lesions.

## AMPK signaling delays the aging process

There is convincing evidence that AMPK signaling controls an integrated signaling network which involves a number of well-known anti-aging pathways, such as the FoxO, NRF2, SIRT1, and ULK1 pathways (Salminen and Kaarniranta 2012; Ge et al. 2022). Moreover, it is known that the activation of AMPK signaling inhibits the function of the NF-κB pathway, which is a major regulator of inflammatory responses (Salminen et al. 2011). There are several reports indicating that AMPK signaling not only extends the lifespan of *Caenorhabditis elegans* (Curtis et al. 2006; Schwarz et al. 2015) and *Drosophila* (Stenesen et al. 2013; Ulgherait et al. 2014) but it can also improve the health span and lifespan of mice (Martin-Montalvo et al. 2013). In addition, there are observations indicating that the activation of AMPK signaling is involved in the extension of lifespan in *C. elegans* and flies induced by dietary restriction (Onken and Driscoll 2010; Canto and Auwerx 2011; Stenesen et al. 2013). Currently, there is an on-going debate whether metformin, an activator of AMPK signaling and a clinically used drug for treatment of type 2 diabetes, could be a putative anti-aging drug (Kulkarni et al. 2020; Mohammed et al. 2021; Chen et al. 2022; Triggle et al. 2022). Metformin confers many health benefits, e.g., it improves energy metabolism, enhances autophagy, modulates mitochondrial dynamics, and mitigates cellular senescence (Kulkarni et al. 2020; Chen et al. 2022), i.e., metformin is able to attenuate many of the typical hallmarks of the aging process. However, the evidence for the role of a chronic exposure to metformin on the lifespan of healthy animals is controversial although there are clear indications that metformin can reduce the mortality associated with various diseases, such as diabetes, cardiovascular disease, and many cancers (Mohammed et al. 2021; Triggle et al. 2022). Currently, it is not known whether an increase in AMPK signaling could prolong the duration of the healthy aging process. It is believed that increased autophagy and an improved mitochondrial function are the major mechanisms enhancing healthy aging by AMPK signaling. However, as described below, AMPK signaling attenuates the differentiation of myofibroblasts, controls the remodeling of extracellular matrix (ECM), reduces the level of fibrotic lesions, and inhibits the activity of immunosuppressive cells, all of which are processes associated with the aging process.

## Myofibroblasts

The properties and functions of myofibroblasts have been described in several extensive review articles (Hinz 2016; Plikus et al. 2021; Schuster et al. 2023). Briefly, myofibroblasts are spindle-shaped contractile fibroblasts which contain a variable amount of stress fibers associated with α-smooth muscle actin proteins (α-SMA). The α-SMA proteins have an important role in the formation of mature focal adhesion (FA) sites which not only facilitate cellular adhesion but also mediate mechanosensing signaling from the ECM to myofibroblasts (Hinz et al. 2003; Kechagia et al. 2019; D’Urso and Kurniawan 2020). Ruffled membranes, a high level of exocytotic vesicles, and an active endoplasmic reticulum are also common characteristics of myofibroblasts. In fact, myofibroblasts represent an activated phenotype of fibroblasts which maintain the structural integrity of connective tissues as well as stromal structures within tissues. Thus, myofibroblasts secrete most of the components of the ECM, such as collagens, fibronectin, and elastin, as well as the proteolytic enzymes which can remodel the structures of the ECM. Fibroblasts display an impressive plasticity, i.e., they can modify their phenotypes in response to changes in the tissue microenvironment (LeBleu and Neilson 2020; Shaw and Rognoni 2020; Bryce et al. 2022; Salminen 2023a). For instance, it has been characterized many heterogeneous subgroups of fibroblasts, e.g., cancer-associated fibroblasts (CAF), fibrosis-associated fibroblasts (FAF), wound-associated fibroblasts (WAF), and aging-associated fibroblasts (AAF) (LeBleu and Neilson 2020). It should be noted that not all subsets of these groups display an increased expression and secretion of fibrous components, e.g., α-SMA and collagens. For example, some subsets express an enhanced level of fibroblast activation protein (FAP) which has been associated with the progression of cancers and rheumatoid arthritis (Fitzgerald and Weiner 2020).

### Myofibroblast differentiation

Single-cell transcriptome experiments have revealed that there exists a substantial diversity between tissue-resident fibroblast populations with respect to their location within tissues as well as between different tissues, species, and age groups (Salzer et al. 2018; Buechler et al. 2021; Plikus et al. 2021). In pathological conditions, chemical or physical insults trigger the differentiation of tissue-resident fibroblasts towards the myofibroblastic state in an attempt to maintain tissue homeostasis and to initiate a repair process. Interestingly, it is not only tissue-resident fibroblasts but many other cell types, especially those of a mesenchymal origin, are able to transdifferentiate into fibrogenic myofibroblasts (LeBleu and Neilson 2020; Plikus et al. 2021). For instance, in fibrotic conditions and cancers, certain fate-mapping studies have revealed that the recruited monocytes and macrophages had become transdifferentiated into myofibroblasts (Meng et al. 2016; Vierhout et al. 2021). The transition of tissue macrophages into fibrogenic myofibroblasts has an especially crucial role in the progression of fibrosis and wound healing (Meng et al. 2016; Tang et al. 2018). Adipocytes, which are also mesenchyme-derived cells, are a significant source of myofibroblast conversion in many fibrotic states, e.g., in efficient skin repair (Shook et al. 2020). It has been claimed that the adipocyte-myofibroblast conversion has an important role in the aging process of facial skin (Kruglikov and Scherer 2016; Wollina et al. 2017). There is substantial evidence that the vascular cells, i.e., pericytes, endothelial cells, and smooth muscle cells, can become converted into myofibroblasts, e.g., in idiopathic pulmonary fibrosis, systemic sclerosis, and hypoxia-induced vascular injuries (Sava et al. 2017; Castellano et al. 2018; van Caam et al. 2018). Fibrocytes are the bone marrow (BM)-derived mesenchymal progenitors which are released from the BM into the circulation and are subsequently recruited into inflamed and fibrotic tissues. In affected tissues, fibrocytes can be differentiated into myofibroblasts, e.g., in bleomycin-induced pulmonary fibrosis in mice (Ashley et al. 2017) and in mouse wounded skin (Mori et al. 2005). Interestingly, there is robust evidence that the differentiated myofibroblasts are able to revert back to their original phenotypes (Hecker et al. 2011; Yang et al. 2014; Fortier et al. 2021). However, occasionally the dedifferentiation capacity of myofibroblasts can be impaired, e.g., in mouse idiopathic pulmonary fibrosis, and this failure leads to the accumulation of fibrotic lesions (Kato et al. 2020). These results highlight the impressive plasticity of fibroblasts which augments the heterogeneity of fibroblast populations in pathological conditions.

While there is convincing evidence that the differentiation and transdifferentiation processes of myofibroblasts are under epigenetic regulation, such as DNA methylation and noncoding RNAs (Hu et al. 2010; Mann et al. 2010; Zhang et al. 2023), the fundamental epigenetic mechanisms are currently poorly understood. However, it is known that there is a wide variety of mechanical and physical inducers and signaling pathways capable of triggering the differentiation of myofibroblasts (D’Urso and Kurniawan 2020; Pakshir et al. 2020). Transforming growth factor-β (TGF-β), a cytokine linked with the SMAD signaling axis, has been demonstrated to be a major inducer of myofibroblast phenotype in several experimental models (Zhan and Kanwar 2014; Vallee and Lecarpentier 2019; Lodyga and Hinz 2020). This pathway involves both the activating Smad2, Smad3, and Smad4 transcription factors and their inhibitory Smad6 and Smad7 components. For instance, Wang et al. (2016) demonstrated that TGF-β/Smad3 signaling promoted the transition of the BM-derived monocytes/macrophages into myofibroblasts in mouse ureteric obstruction-induced kidney fibrosis. The TGF-β/Smad3 pathway stimulates the expression of NADPH oxidases (NOX) which promote ROS production in tissues. It is known that the TGF-β-induced reactive oxygen species (ROS) are effective inducers of myofibroblast differentiation and thus are associated with the appearance of fibrotic lesions (Barnes and Gorin 2011). In fact, it has been reported that ROS were able to activate the NLRP3 inflammasomes which subsequently enhanced myofibroblast differentiation and induced fibrosis in diverse experimental models (Alyaseer et al. 2020; Ji et al. 2021). TGF-β signaling can also stimulate myofibroblast transition by increasing the amount of collagen cross-links and thus augmenting the stiffness of the ECM (Cho et al. 2018; D’Urso and Kurniawan 2020). There exists a number of other signaling mechanisms, co-operating either with TGF-β/SMAD signaling or not, which are able to induce the conversion of myofibroblasts in diverse setups. For instance, it is known that the activation of the NF-κB/NLRP3 axis (Boaru et al. 2015; Ji et al. 2021; Lopez-Antona et al. 2022), the JAK/STAT3 pathway (Pedroza et al. 2016; Chakraborty et al. 2017), the Yap/Taz factors (He et al. 2022), and the non-canonical Wnt pathway (Liu et al. 2021), can induce the differentiation of myofibroblasts. The versatile signaling mechanisms in the differentiation of myofibroblasts support the plasticity of fibroblast populations within tissues.

### Fibrogenic and immune properties of myofibroblasts

The accumulation of myofibroblasts and their robust activation are typically associated with acute tissue injuries, e.g., myocardial infarction, although chronic inflammatory states can also induce an excessive deposition of fibrotic lesions in different pathological states, such as in idiopathic pulmonary fibrosis, atherosclerosis, arthrofibrosis, systemic sclerosis, and subretinal fibrosis in age-related macular degeneration (Weiskirchen et al. 2019; Talbott et al. 2022; Tenbrock et al. 2022; Schuster et al. 2023). It is not only mesenchymal tissue-resident fibroblasts which can differentiate myofibroblasts during fibrotic states but the transdifferentiation of inflammatory macrophages into myofibroblasts plays an important role in inflammatory states, such as in renal and subretinal fibrosis (Meng et al. 2016; Little et al. 2020). In addition, many immunosuppressive cells, e.g., myeloid-derived suppressor cells (MDSC) and regulatory T cells (Treg), have a crucial role in the pathogenesis of fibrotic diseases (Huaux 2021; van Geffen et al. 2021). Currently, there is convincing evidence that tissue fibrosis can be a reversible process, i.e., fibrogenic myofibroblasts can be eliminated and fibrotic ECM can be cleansed (Jun and Lau 2018; Horowitz and Thannickal 2019). There exists an increased number of senescent fibroblasts in fibrotic tissues and currently, it seems that pro-inflammatory senescent fibroblasts might aggravate fibrosis rather than restrict the expansion of fibrotic lesions (Hernandez-Gonzalez et al. 2021).

Fibroblasts act as versatile immune regulators collaborating with the cells of immune system in an attempt to maintain the homeostasis of tissues (Van Linthout et al. 2014; Mhaidly and Mechta-Grigoriou 2020; Davidson et al. 2021; Huaux 2021; Schuster et al. 2021). The fibroblast-immune cell interactions are highly context-dependent with respect to tissue types and the pathological states (Krausgruber et al. 2020; Davidson et al. 2021). Interestingly, fibroblasts/myofibroblast are able to display both pro- and anti-inflammatory responses involving the secretion of cytokines, chemokines, growth factors, and complement components as well as they can express ligands and receptors for immune checkpoint proteins (Powell et al. 1999; Van Linthout et al. 2014; Zhao et al. 2023). Cancer associated fibroblasts (CAF) can display different immune phenotypes, such as the inflammatory CAFs (iCAF), the immunosuppressive myofibroblast population (myCAF), the antigen-presenting CAFs (apCAF), and the complement-secreting CAFs (csCAF) (Costa et al. 2018; Mhaidly and Mechta-Grigoriou 2020; Bryce et al. 2022; Ngwenyama et al. 2022). For instance, during mouse myocardial infarction there occurred a transition of inflammatory fibroblasts to anti-inflammatory/immunosuppressive myofibroblast population with fibrogenic properties (Daseke et al. 2020). In particular, myofibroblasts have many close interactions with immunosuppressive cells, such as MDSCs, Tregs, and M2 polarized macrophages. This can be attributed to the presence of TGF-β and certain other cytokines and chemokines which have reciprocal effects on both myofibroblasts and immunosuppressive cells (Monteran and Erez 2019; Mhaidly and Mechta-Grigoriou 2020; Mao et al. 2021; Salminen 2023b). For example, TGF-β secreted by immunosuppressive cells stimulates the differentiation of myofibroblasts, whereas TGF-β, IL-6, IL-10, and colony-stimulating factors (CSF) as well as many chemokines released by myofibroblasts (Powell et al. 1999) are potent inducers of both the recruitment and activation of immunosuppressive cells. Moreover, as discussed above, TGF-β signaling can promote the transdifferentiation of macrophages into myofibroblasts, e.g., in fibrotic microenvironments. The M2 polarized macrophages secrete TGF-β and IL-10 in chronic inflammatory diseases and thus they not only promote myofibroblast differentiation but they also enhance the activation of immunosuppressive cells. Disturbances in the intricate crosstalk between myofibroblasts and immunosuppressive cells seem to be evident in many chronic diseases, especially cancers and fibrotic lesions in many inflammatory diseases.

### Myofibroblasts in the aging process

The structural changes in the ECM are a common hallmark of the aging process (Freitas-Rodrigues et al. 2017; Angelini et al. 2020; Selman and Pardo 2021). For instance, there is an increase in the degree of crosslinking between collagen proteins which augments the stiffness of the ECM with aging. As described earlier, this enhanced stiffness stimulates the differentiation of myofibroblasts and promotes tissue fibrosis. Fibrotic lesions are commonly observed in aged tissues, such as in myocardium, lungs, and kidney (Biernacka and Frangogiannis 2011; Xu et al. 2020; Selman and Pardo 2021). In particular, the level of fibrotic lesions is elevated in aging-associated chronic diseases. The age-related collagen crosslinking and matrix stiffness not only enhance the differentiation of myofibroblasts but they also promote the activation of immunosuppressive cells, such as M2 macrophages (Xing et al. 2021), Tregs (Shi et al. 2023), and MDSC (Chiodoni et al. 2017). It is known that there are increases in the presence and activity of immunosuppressive cells with aging both in the circulation and within tissues (Salminen 2020). Immunosuppressive cells have a close interaction with myofibroblasts and this crosstalk seems to promote the accumulation of fibrotic lesions, especially in inflammatory conditions (Mhaidly and Mechta-Grigoriou 2020; Huaux 2021; Mao et al. 2021; van Geffen et al. 2021; Salminen 2023b). With respect to the aging process, it seems that cellular senescence displaying the pro-inflammatory senescence-associated secretory phenotype (SASP) is a major source of chronic low-grade inflammation (Campisi 2013; Franceschi et al. 2018). There is robust evidence that inflammatory mediators are potent inducers of the myofibroblast differentiation process (see below) which indicates that the age-related fibrosis represents one mechanism intended to counteract the presence of a chronic inflammatory state. Currently, only a few investigators have applied the single-cell transcriptomics to examine the age-related changes in the fibroblast population within different tissues (Salzer et al. 2018; Vidal et al. 2019; Sole-Boldo et al. 2020). Salzer et al. (2018) demonstrated that with aging, mouse dermal fibroblasts partially lost their identity and acquired certain adipogenic traits. Sole-Boldo et al. (2020) also revealed a gradual loss of cellular identity with aging in a human skin fibroblast population. They reported that aging reduced many functional properties, e.g., the proliferation activity decreased, whereas the expression of certain pro-inflammatory genes was clearly up-regulated. These single-cell studies indicated that the heterogeneity of fibroblast subpopulations declines with aging, probably might be attributed to the senescence of fibroblasts. Currently, single-cell studies have been limited to a few tissues, especially to skin, and only certain properties, such as SASP factors, have been characterized in a more detailed manner.

In addition to collagen crosslinking and matrix stiffness, several other age-related changes present in tissues can trigger the appearance of myofibroblasts and enhance their survival leading to fibrotic lesions in aged tissues. For instance, oxidative stress, a hallmark of the aging process, has been demonstrated to be a potent inducer of the myofibroblast differentiation in several experimental models (Toullec et al. 2010; Taddei et al. 2012; Montorfano et al. 2014; Shimura et al. 2018). However, ROS are complex regulators since they are connected to a number of important upstream and downstream signaling pathways. It does seem that the ROS production via the TGF-β/Smad1/NADPH oxidase 4 (NOX4) has a crucial role in the differentiation of myofibroblasts in many pathological conditions (Hecker et al. 2009; Bondi et al. 2010; Barnes and Gorin 2011). Bondi et al. (2010) demonstrated that the NOX4-induced ROS production stimulated the MEK/ERK1/2 kinase pathway which in turn induced the expression of α-SMA protein and promoted myofibroblast differentiation in mouse kidney fibroblasts. Oxidative stress is also able to stimulate the generation of advanced glycation endproducts (AGE) which induce myofibroblast differentiation via the AGE/RAGE signaling pathway (Oldfield et al. 2001). Accordingly, the high mobility group box 1 (HMGB1), a ligand for RAGE and an enhancer of inflammation, was able to convert human lung fibroblasts into myofibroblasts (Lee et al. 2015). There are observations indicating that endoplasmic reticulum stress, also competent to generate ROS, stimulated myofibroblast differentiation and accordingly this promoted the accumulation of fibrotic lesions (Baek et al. 2012; Jiang et al. 2018). Moreover, ROS are potent activators of NLRP3 inflammasomes (Tschopp and Schroder 2010) which are known to stimulate the differentiation of myofibroblasts and augment tissue fibrosis (Ji et al. 2021; Artlett 2022). The mechanism still needs to be clarified although it is known that IL-18, a product of NLRP3 activation, promoted tissue fibrosis (Zhou et al. 2016; Artlett 2022). Moreover, several other inflammatory cytokines are also able to activate the differentiation of myofibroblasts and enhance fibrosis in several experimental models (Van Linthout et al. 2014; van Caam et al. 2018). Given that the aging process is associated with chronic low-grade inflammation, it seems plausible that the inflammation-induced production of ROS compounds and other inflammatory mediators are major inducers of myofibroblast differentiation and subsequently migh promote the accumulation of fibrotic lesions.

## AMPK signaling inhibits the differentiation of myofibroblasts

**Fig. 1:**
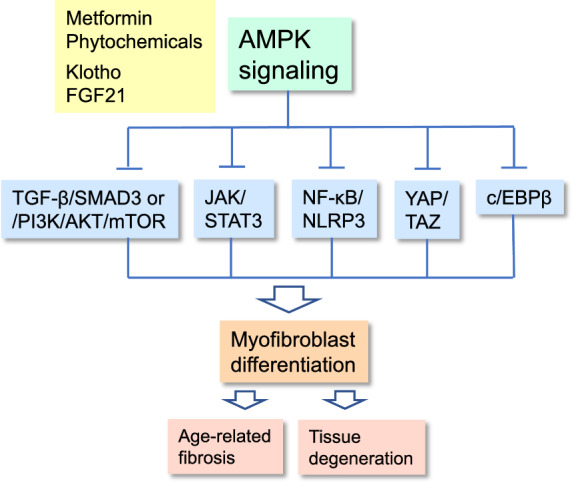
Schematic presentation depicting the signaling connections through which AMPK signaling inhibits the differentiation of myofibroblasts. TGF-β-driven pathways, JAK/STAT3, NF-κB/NLRP3, YAP/TAZ, and c/EBPβ signaling are the major pathways stimulating myofibroblast differentiation which subsequently promotes age-related fibrosis and tissue degeneration. Treatments with metformin or certain phytochemicals as well as increased endocrine levels of klotho and FGF21 proteins activate AMPK signaling and thus attenuate myofibroblast differentiation and subsequently alleviate the accumulation of age-related fibrotic lesions. Stoppers indicate inhibitory connections and an arrow denotes the activation of myofibroblast differentiation. Abbreviations: AKT, protein kinase B; AMPK, AMP-activated protein kinase; c/EBPβ, CCAAT-enhancer-binding protein β; FGF21, fibroblast growth factor 21; JAK; Janus kinase; mTOR, mammalian target of rapamycin; NF-κB, nuclear factor-κB; NLRP3, NLR family pyrin domain containing 3; PI3K, phosphoinositide 3-kinase; Smad3, SMAD family member 3; STAT3, signal transducer and activator of transcription 3; TAZ, transcriptional coactivator with PDZ-binding motif; TGF-β, transforming growth factor-β; YAP, Yes-associated protein

The activation of tissue-resident fibroblasts as well as the transdifferentiation of non-fibroblasts into myofibroblasts are regulated by diverse signaling pathways. Interestingly, it seems that AMPK signaling is a potent inhibitor of this versatile regulatory network, thus attenuating tissue fibrosis and immunosuppression associated with many chronic diseases (Fig. 1).

### TGF-β-driven Smad2/3 and PI3K/AKT/mTOR pathways

The TGF-β/Smad2/3 signaling pathway is the main inducer of myofibroblast differentiation and it acts in a close co-operation with immunosuppressive cells, as described above (Lodyga and Hinz 2020; Mhaidly and Mechta-Grigoriou 2020; Huaux 2021; Mao et al. 2021). Interestingly, there are many investigations conducted in several experimental models indicating that the activation of AMPK signaling is a potent inhibitor of the TGF-β-driven differentiation of myofibroblasts, efficiently preventing tissue fibrosis (Mishra et al. 2008; Lin et al. 2015; Thakur et al. 2015) (Fig. 1). Mishra et al. (2008) demonstrated that the activation of AMPK signaling with 5-amino-1-(5-phospho-D-ribosyl)imidazole-4-carboxamide (AICAR) and adiponectin inhibited the TGF-β-induced conversion of human renal mesangial cells into myofibroblasts. They also revealed that the suppression of myofibroblast transition was dependent on the AMPKα2-induced inhibition of transcriptional activity of Smad3 factor which acted as a driving force for myofibroblast differentiation. Lin et al. (2015) reported that AMPK signaling repressed the TGF-β-induced epithelial-to-mesenchymal transition and myofibroblast activation via inhibiting the phosphorylation and transcriptional activation of Smad2/3 factors in human bronchial epithelial cell line. Taken together, these studies indicated that AMPK signaling was able to suppress the Smad2/3-driven transcription and subsequently inhibit myofibroblast activation and tissue fibrosis.

In addition to the Smad2/3 pathway, it seems that there also exist other inhibitory mechanisms driven by AMPK signaling. For instance, there is clear evidence indicating that TGF-β treatment can trigger myofibroblast differentiation via the PI3K/AKT/mTOR signaling pathway (Tang et al. 2023; Chen et al. 2023). For instance, Chen et al. (2023) demonstrated that exposure to TGF-β induced the conversion of mouse renal pericytes into myofibroblasts. The TGF-β treatment of pericytes robustly increased the expression of hexokinase II which enhanced the amount of glycolysis in converted myofibroblasts. It is known that aerobic glycolysis is the major energy metabolic pathway utilized by myofibroblasts (Xie et al. 2015). Chen et al. (2023) also reported that the inhibition of either glycolytic flux or the activity of PI3K/AKT/mTOR signaling suppressed the conversion of the TGF-β-stimulated myofibroblasts, indicating that this pathway is an alternative route for myofibroblast differentiation. Moreover, Tang et al. (2023) revealed that the TGF-β-induced PI3K/AKT/mTOR signaling induced the differentiation of canine valvular interstitial cells into myofibroblasts which displayed a phenotype with a reduced capacity for autophagy and an increase in senescence-associated secretory functions. There is abundant evidence that mTOR is a potent inhibitor of autophagy, whereas AMPK signaling inhibits the function of mTOR and augments autophagy (Kim et al. 2011; Alers et al. 2012). It is known that mTOR signaling has an important role in the promotion of the aging process and age-related diseases (Liu and Sabatini 2020). Furthermore, Ko et al. (2016) reported that rapamycin, a specific inhibitor of mTOR, suppressed the TGF-β-induced transition of human nasal fibroblasts into myofibroblasts via the PI3K/AKT/mTOR pathway. These observations indicate that mTOR signaling has a crucial role in the differentiation of myofibroblasts. However, the mTOR-driven signaling is a complex nexus of different activities and currently the roles of AMPK signaling and autophagy in the mTOR-dependent differentiation of myofibroblast remain to be clarified.

### JAK/STAT3 pathway

There is substantial evidence that the JAK/STAT3 pathway has a crucial role in many fibrotic diseases, e.g., systemic sclerosis, pulmonary fibrosis, and renal fibrosis (Pedroza et al. 2016; Chakraborty et al. 2017; Liu et al. 2023). Currently, STAT3 inhibitors are promising drug candidates for different fibrotic diseases (Wang et al. 2023a, b; Xie et al. 2023). JAK/STAT3 signaling has been associated with the activation of several cytokine receptors, such as those of IL-4, IL-6, IL-10, GM-CSF, as well as many growth factor receptors. For instance, Tang et al. (2017) demonstrated that TGF-β induced mouse hepatic fibrosis in a close coordination between the JAK1/STAT3 axis and the SMAD pathway. The JAK/STAT3 axis has also a major role in the activation of immunosuppressive cells, such as MDSCs and Tregs. Interestingly, it is known that many regulatory immune cells, e.g., MDSCs and Tregs, augment the pathogenesis of fibrotic diseases (Huang et al. 2020; van Geffen et al. 2021), probably in co-operation with myofibroblasts. There are diverse studies indicating that the activation of the JAK/STAT3 axis promotes the differentiation of myofibroblasts in many experimental models (Pedroza et al. 2016; Chakraborty et al. 2017; Milara et al. 2018) (Fig. 1). The JAK2-induced STAT3 phosphorylation is an especially potent inducer of myofibroblast differentiation in human and mouse pulmonary fibrosis (Milara et al. 2018). Pedroza et al. (2016) demonstrated that the level of the activated phospho-STAT3 was robustly increased in both human fibrotic lungs and mouse bleomycin-induced lung fibrosis. They also revealed that the inhibition of STAT3 activation reduced myofibroblast differentiation as well as the severity of mouse pulmonary fibrosis. Chen et al. (2014) reported that in mouse kidney fibroblasts the silencing of AMPKα1 expression or the exposure to compound C, a cell-permeable inhibitor of AMPK activity, prevented inhibitory effects of AICAR on the TGF-β-induced myofibroblast phenotype. The AICAR treatment also reduced the severity of renal fibrosis and the deposition of ECM components in an obstructed kidney model. Moreover, Chen et al. (2014) demonstrated that AICAR exposure prevented the TGF-β-induced myofibroblast differentiation via the down-regulation of STAT3 signaling. It is known that AMPK signaling is a potent inhibitor of the function of the JAK/STAT3 pathway (Nersted et al. 2010; Rutherford et al. 2016). For instance, Ji et al. (2023) revealed that the metformin-induced AMPK signaling clearly inhibited the level of phospho-STAT3 and reduced myofibroblast differentiation in mouse bleomycin-induced pulmonary fibrosis. Given that AMPK signaling also inhibits the function of MDSCs via the inhibition of JAK/STAT3 signaling (Salminen et al. 2019), it seems that inhibitors of JAKs (jakinibs) and STAT3 (stattics) could alleviate fibrotic lesions by inhibiting the differentiation of myofibroblasts and attenuating their collaboration with immunosuppressive cells.

### NF-κB/NLRP3 axis

The NF-κB signaling pathway not only regulates immune responses but it can also promote the differentiation of fibroblasts into myofibroblasts and subsequently it enhances their survival which in turn aggravates the severity of fibrotic diseases (Watson et al. 2008; Oakley et al. 2009; Hou et al. 2018). The NF-κB signaling system interacts with many other signaling pathways, e.g., the TGF-β/Smad3 and JAK/STAT3 pathways. Interestingly, it seems that NF-κB signaling induces myofibroblast differentiation through the activation of inflammasomes, especially the NLR family pyrin domain containing 3 (NLRP3) inflammasomes (Kuang et al. 2018) (Fig. 1). For example, NF-κB signaling stimulates the priming of the NLRP3 inflammasomes, i.e., it stimulates the expression of proforms of IL-1β and IL-18 cytokines as well as the caspase-1 protein which is a triggering enzyme in the activation of the NLRP3 inflammasomes (Boaru et al. 2015; Kelley et al. 2019). The activation of the NLRP3 inflammasomes generates mature IL-1β and IL-18 cytokines which are subsequently secreted from cells. There are several studies indicating that the activation of NLRP3 inflammasomes stimulates the differentiation of myofibroblasts and promotes fibrosis in many experimental models (Artlett et al. 2011; Alyaseer et al. 2020; Ji et al. 2021). For instance, Artlett et al. (2011) reported that the NLRP3 knockout mice were resistant to the bleomycin-induced skin fibrosis. Interestingly, it seems that it is the mature IL-18 cytokine which promotes myofibroblast differentiation and fibrosis (Zhou et al. 2016; Liang et al. 2018), since it is known that IL-1β, another inflammasome product, suppresses the TGF-β-induced myofibroblast formation from human dermal and lung fibroblasts (Mia et al. 2014) and also from rat cardiac fibroblasts (Bronnum et al. 2013).

There are several reports indicating that AMPK signaling inhibits the activation of NLRP3 inflammasomes via different mechanisms (Cordero et al. 2018; Yang et al. 2019; Dong et al. 2020) (Fig. 1). It seems that AMPK signaling can inhibit the function of NLRP3 inflammasomes by controlling autophagy, mitochondrial function, and the activity of SIRT1 deacetylase. For instance, Li et al. (2017) reported that an increase in the activity of SIRT1 signaling inhibited the function of NLRP3 inflammasomes in human vascular endothelial cells. Moreover, several phytochemicals, such as artemisinin and curcumin, have been shown to suppress the activity of NLRP3 inflammasomes via the stimulation of AMPK signaling (Li et al. 2015; Jiang et al. 2020). Jiang et al. (2020) revealed that exposure to artemisinin ameliorated the severity of fibrotic lesions in mouse atherosclerotic aorta by reducing macrophage activation via the AMPK/NF-κB/NLRP3 pathway. In addition, some hormones, e.g., adiponectin and relaxin, are able to activate AMPK signaling (Chen et al. 2013; Aragon-Herrera et al. 2018) and accordingly, it is known that these hormones are able to inhibit the activation of NLRP3 inflammasomes and consequently they attenuated the degree of fibrosis (Dong et al. 2020; Tapia Caceres et al. 2022). Currently, it seems likely that AMPK signaling suppresses the NLRP3-induced differentiation of myofibroblast and attenuates tissue fibrosis although the molecular mechanisms need to be clarified.

### YAP/TAZ signaling

The Yes-associated protein (YAP) and the transcription coactivator with the PDZ-binding motif (TAZ) are two transcriptional activators which enhance the activity of the members of the TEA domain transcription factor (TEAD) family (Heng et al. 2021). The YAP/TAZ factors are also able to regulate the function of some other signaling pathways, e.g., the TGF-β/Smad3 pathway (Szeto et al. 2016; Hu et al. 2021). It is known that the YAP/TAZ factors regulate many developmental processes, such as the development of muscular tissues, as well as participating in cell proliferation, apoptosis, and many immune functions. Several mechanical insults, such as ECM stiffness, tissue stretching, and fluid shear stress, stimulate the YAP/TAZ signaling pathway which subsequently enhances the expression of many contractile proteins, such as the α-SMA protein (Cai et al. 2021). There is abundant evidence indicating that the YAP/TAZ factors have a crucial role in the differentiation of myofibroblasts and production of fibrotic lesions in diverse experimental models (Muppala et al. 2019; Hu et al. 2021; Ragazzini et al. 2022; Xu et al. 2022). Hu et al. (2021) demonstrated that the mechanical strain of human scleral fibroblasts promoted the expression of YAP and induced the differentiation of fibroblasts into myofibroblasts. Accordingly, a knockdown of YAP expression reduced the mechanical strain-induced α-SMA expression and myofibroblast differentiation. These investigators also reported that the YAP protein interacted with the Smad3 factor to induce a myofibroblast transition, whereas the inhibition of Smad3 signaling blocked the conversion of myofibroblasts. Both the Integrin/FAK/Src (Ma et al. 2020) and the RhoA/ROCK (He et al. 2019) signaling mechanisms can trigger mechanotransduction which stimulates myofibroblast differentiation and augments tissue fibrosis. Currently, it is known that the activation of YAP/TAZ signaling has a major role in development of fibrotic diseases (Mia and Singh 2022).

Energy supply of myofibroblasts is dependent on aerobic glycolysis (Xie et al. 2015) and accordingly an inhibition of glycolysis suppressed the differentiation of myofibroblasts and attenuated tissue fibrosis (Xie et al. 2015; Ding et al. 2017; Chen et al. 2021). There are several studies indicating that a deficiency in the cellular energy supply induced an AMPK-dependent phosphorylation of the YAP protein which subsequently caused an inhibition of the YAP-driven transcription (DeRan et al. 2014; Mo et al. 2015; Wang et al. 2015) (Fig. 1). AMPK signaling induced the phosphorylation of the YAP protein at many sites, e.g., at Ser61, Ser94, and Ser127, which impaired its transport into nuclei or inhibited its binding to the TEAD factor. Furthermore, the phosphorylation of the YAP protein enhanced its degradation in proteasomes. It is also known that the inhibition of glycolysis could prevent the accumulation of fibrotic lesions after tissue injuries. For example, Chen et al. (2021) demonstrated in mice that a blockade of glycolytic flux after an experimental myocardial infarction suppressed myofibroblast differentiation and alleviated the severity of cardiac fibrosis. Given that the function of myofibroblasts, e.g., an increase in contractility and enhanced synthesis of ECM proteins, requires efficient energy production, it seems that modulating AMPK/YAP signaling would represent a promising therapeutic approach to achieving an inhibition of myofibroblast formation.

### Other pathways

There are observations indicating that AMPK signaling could also inhibit myofibroblast differentiation and fibrosis via certain other pathways although their involvement needs to be clarified. For instance, endoplasmic reticulum (ER) stress augmented the differentiation of myofibroblasts (Baek et al. 2012) and there are studies showing that AMPK signaling alleviated ER stress and renal fibrosis in mice (Kim et al. 2015). Yang et al. (2022) reported that ER stress stimulated the expression of C/EBP homologous protein (CHOP) which subsequently was able to convert mouse mesenchymal stem cells into myofibroblasts. They also revealed that CHOP signaling promoted the function of the TGF-β/SMAD axis and in that way, it probably enhanced myofibroblast transition and augmented pulmonary fibrosis. Dai et al. (2016) demonstrated in mouse macrophages that AMPKα1 signaling phosphorylated the CHOP protein which promoted its degradation in proteasomes. This implies that AMPK signaling can attenuate the CHOP-mediated differentiation pathway to myofibroblast. Many other signaling mechanisms can potentially enhance the transition of myofibroblasts but there are contradictory results, i.e., it is not known whether or not these pathways are inhibited by AMPK signaling. For example, the activation of glycogen synthase kinase-3α/β (GSK-3α/β) augmented the differentiation of myofibroblasts in different fibrotic states (Baarsma et al. 2013; Singh et al. 2015) but it did seem that the effects of AMPK signaling on the GSK-3α/β activity were dependent on the experimental conditions. In addition, it is known that the WNT/β-catenin pathway is a potent inducer of myofibroblast differentiation and an enhancer of tissue fibrosis (Piersma et al. 2015; Dzialo et al. 2021). However, it has close interactions with the TGF-β and the YAP/TAZ pathways which are inhibited by AMPK signaling, as described above. It seems that AMPK signaling inhibits fibrosis in a tissue-specific manner since there exist robust differences between tissues in the activities of the signaling pathways promoting fibrosis.

## AMPK signaling inhibits fibrotic lesions and tissue degeneration

There is a general consensus that it is the differentiation of myofibroblasts, either from tissue-inherent fibroblasts and/or from other cell types via transdifferentiation, that is the key mechanism in the pathogenesis of tissue fibrosis (Frangogiannis 2019; Pakshir et al. 2020; Lurje et al. 2023; Schuster et al. 2023). Tissue injuries, in either acute or chronic forms, are major triggers of profibrotic processes promoting tissue fibrosis. Inflammation and the close co-operation between the many immune cells are important enhancers of the accumulation of fibrotic lesions in tissues. Thus, it is not surprising that there is significant therapeutic interest in clarifying the signaling mechanisms which drive and support fibrogenic processes in tissues. The last decade has seen important progress in our understanding of these profibrotic signaling mechanisms (see above) although single-cell sequencing experiments have revealed that fibrosis is a rather heterogeneous process with respect to changes in cell populations (Adams et al. 2020; Deng et al. 2021). Currently, there is robust evidence that AMPK signaling is able to attenuate the severity of fibrotic lesions in tissues in many experimental models and also in certain fibrotic diseases (Daskalopoulos et al. 2016; Jiang et al. 2017; Liang et al. 2017). As discussed above, different agonists of AMPK activation, such as (1) AICAR and metformin, (2) hormonal adiponectin and relaxin, as well as (3) certain phytochemicals, have been able to inhibit the myofibroblast transition and subsequently lessen the deposition of fibrotic lesions in diverse experimental models. On the other hand, there are studies indicating that it is the resistance to apoptosis attributable to myofibroblasts that actually promotes the deposition of fibrotic lesions within tissues (Thannickal and Horowitz 2006; Hinz and Lagares 2020). Interestingly, it does seem that AMPK signaling can stimulate cellular apoptosis via different mechanisms (Day et al. 2011; Rangarajan et al. 2018). For instance, Rangarajan et al. (2018) demonstrated that metformin exposure reversed the bleomycin-induced fibrosis in mice by increasing the apoptosis of fibroblasts in an AMPK-dependent manner. Currently, there are intensive drug discovery projects aimed to attack tissue fibrosis either by inhibiting myofibroblast transition or enhancing fibroblast apoptosis in fibrotic tissues. Recently, Zhao et al. (2022) listed all of the current drugs for fibrotic diseases, either marketed or under clinical trials. Only four drugs have received marketing approval, i.e., pirfenidone and nintedanib for idiopathic pulmonary fibrosis and ruxolitinib and panobinostat for myelofibrosis. It is claimed that pirfenidone targets TGF-β signaling, whereas ruxolitinib acts on the JAK1/2-driven pathways. However, there are several drug candidates targeting in a direct or indirect manner the signaling pathways which are known to be affected by AMPK signaling.

AMPK signaling can attenuate tissue fibrosis not only by suppressing the differentiation of myofibroblasts or enhancing their apoptosis but it can also modulate the functions of immune cells and thus control the co-operation between myofibroblasts and immune cells during the expansion of fibrotic lesions. There is clear evidence that after an injury, not only are pro-inflammatory fibroblasts involved in the recruitment of immunosuppressive cells into inflamed tissues but they also enhance the polarization of M1 macrophages into anti-inflammatory M2 phenotypes in an attempt to counteract the excessive inflammation (see above). However, in chronic inflammatory states, immunosuppressive cells, such as MDSCs, Tregs, and M2 macrophages, promote the development of fibrogenic myofibroblasts and thus aggravate the severity of fibrosis (Huang et al. 2020; Huaux 2021; van Geffen et al. 2021; Hara and Tallquist 2023). Immunosuppressive cells secrete many anti-inflammatory cytokines, such as TGF-β, IL-6, and IL-10, which are known to be potent enhancers of the myofibroblast transition. There are many reports indicating that the numbers of MDSCs are increased in both human and mouse peripheral blood and lung tissues in pulmonary fibrosis (Fernandez et al. 2016; Lebrun et al. 2017; Liu et al. 2022). For instance, Lebrun et al. (2017) demonstrated that monocytic MDSCs (M-MDSC) infiltrated into mouse lungs during the development of silica-induced pulmonary fibrosis. They also revealed that the silica-induced fibrosis was clearly reduced in M-MDSC-deficient mice, i.e., evidence of the important role of MDSCs in the generation of pulmonary fibrosis. In fact, it is known that an activation of AMPK signaling downregulates the immunosuppressive activity of MDSCs (Qin et al. 2018; Li et al. 2018; Salminen et al. 2019; Xu et al. 2019). For instance, Li et al. (2018) revealed that metformin treatment reduced the expression of CD39 and CD73 proteins via the activation of AMPKα in the MDSCs isolated from human ovarian cancer. A decline in the expression of CD39 and CD73 proteins significantly reduced the immune suppressive activity of MDSCs. Although metformin and cancer-related MDSCs were utilized, these observations indicated that it was AMPK signaling that suppressed the function of MDSCs and thus its activation could represent one mechanism to reduce the level of tissue fibrosis.

The results with respect to the AMPK-induced inhibition of Tregs and M1/M2 macrophages in fibrosis are more inconsistent although there is clear evidence that Tregs and M2 macrophages can promote fibrogenic processes (Lo Re et al. 2011; Braga et al. 2015; Birjandi et al. 2016; Little et al. 2020). For instance, Vasamsetti et al. (2015) demonstrated that metformin and AICAR treatments inhibited the differentiation of monocytes into macrophages and accordingly, metformin attenuated the Ang-II-induced formation of atherosclerotic lesions in mice. Recently, Nassif et al. (2022) demonstrated that metformin exposure of human M2 macrophages inhibited the production of ROS via the activation of AMPK signaling. This is an interesting observation since it is known that ROS can aggravate the pathogenesis of many fibrotic diseases, e.g., pulmonary fibrosis (Richter and Kietzmann 2016; Otoupalova et al. 2020; Estornut et al. 2022). Previously, Han et al. (2016) reported that AMPK activation protected human and mouse fibroblasts from the cellular senescence evoked by ROS exposure. As described above, senescent fibroblasts aggravate the expansion of lesions in fibrotic diseases (Hernandez-Gonzalez et al. 2021). In chronic fibrotic states, the existence of an immunosuppressive microenvironment, i.e., an environment containing an increased level of TGF-β and other immunosuppressive mediators secreted by immune cells and myofibroblasts, enhances the degenerative processes in tissues. The presence of TGF-β does not only promote myofibroblast differentiation and the activation of immunosuppressive cells but it also impairs tissue homeostasis (Tominaga and Suzuki 2019; Salminen 2021). For instance, TGF-β signaling (1) arrests the cell cycle, (2) promotes cellular senescence of neighbouring cells, (3) enhances the deposition of ECM components, (4) induces the secretion of matrix metalloproteinases and collagenases, (5) activates the myeloid-biased response in hematopoietic stem cells (HSC), and finally (6) promotes several age-related diseases, such as cardiovascular diseases, sarcopenia, and skin atrophy. Moreover, immunosuppressive cells display a strong expression of arginase (ARG) and indoleamine 2,3-dioxygenase (IDO), two enzymes which deplete the levels of L-arginine and L-tryptophan from the tissue microenvironment (Murray 2016; Mellor et al. 2017). A shortage of these amino acids causes bystander effects in neighboring cells, especially in effector immune cells. In fact, myofibroblast proliferation and tissue fibrosis disturb the maintenance of homeostasis and promote tissue degeneration. Given that AMPK signaling is able to inhibit fibrotic processes, it seems that the anti-aging properties of AMPK signaling may be partly attributed to the inhibition of the tissue fibrosis associated with the aging process.

## Does AMPK signaling alleviate the aging process by inhibiting myofibroblast differentiation?

The presence of fibrotic lesions within tissues is a common hallmark of the aging process (Selman and Pardo 2021; Ren et al. 2023). As discussed above, several age-related molecular changes, such as oxidative and ER stresses as well as profound alterations in the characteristics of the ECM, promote myofibroblast differentiation and subsequently trigger the deposition of fibrotic material into tissues. In fact, it seems that it is a chronic low-grade inflammatory process which stimulates the recruitment of immunosuppressive cells into peripheral tissues in an attempt to counteract the harmful inflammatory environment that is associated with aging. There is mounting evidence that during aging, the number of immunosuppressive cells increases both in the circulation and within peripheral tissues (Salminen 2020). Currently, the origins of the age-related inflammatory processes are unknown although there are several possible sources which could trigger an inflammaging state within tissues. Nonetheless, immunosuppressive cells secrete many compounds, e.g., TGF-β, IL-10, and ROS, which enhance the differentiation of myofibroblasts. Furthermore, myofibroblasts secrete cytokines, growth factors, proteolytic enzymes, and fibrotic materials which are able to remodel the structures of the ECM (Powell et al. 1999). Interestingly, TGF-β and ROS are also potent enhancers of cellular senescence in fibrotic tissues (Tominaga and Suzuki 2019; Parimon et al. 2021). Senescent fibroblasts are pro-inflammatory and pro-fibrotic cells and thus cellular senescence augments fibrosis in tissues (Tigges et al. 2014; Schafer et al. 2017; Hernandez-Gonzalez et al. 2021). Single-cell transcriptome experiments have revealed the impressive heterogeneity in the properties of fibroblast population in aged tissues (Waldera-Lupa et al. 2014; Mahmoudi et al. 2019; Zou et al. 2021). One reason for this heterogeneity could be that with aging the resolution capacity of fibrosis becomes significantly decreased, probably attributable to a decline in their apoptotic susceptibility (Hecker et al. 2014; Kato et al. 2020). Kato et al. (2020) demonstrated that senescent fibroblasts in culture and myofibroblasts from mouse idiopathic pulmonary fibrosis displayed a significant impairment in their resolving activity which was associated with an age-related increase in their resistance to apoptosis. Kato et al. (2020) also revealed that the expression of myoblast determination protein (MyoD) was robustly elevated in myofibroblasts in age-related persistent fibrosis and accordingly the knockdown of MyoD protein restored the susceptibility of myofibroblasts to apoptosis and also enhanced the resolution of fibrosis. It seems that the co-operation between myofibroblasts and immunosuppressive cells not only inhibits chronic low-grade inflammation but also promotes degenerative processes in tissues, e.g., via increased cellular stress, decreased autophagic activity, and the accumulation of fibrotic lesions.

As described above, there is convincing evidence that AMPK signaling inhibits the differentiation of fibroblasts and several other cell types into myofibroblasts. Given that metformin and AICAR are not specific for AMPK signaling, many investigators have confirmed the specificity of AMPK signaling using knockout and knockdown technologies as well as the inhibitors of AMPK activity. Currently, certain activators of AMPK signaling, e.g., metformin, are claimed to be potential anti-aging compounds since these compounds are able to extend health span and even lifespan in rodents (see above). There are also certain endocrine factors, such as the klotho protein and fibroblast growth factor 21 (FGF21), which are recognized as anti-aging factors since they can attenuate many age-related degenerative processes, e.g., in the vascular system (Kurosu et al. 2005; Mencke et al. 2017a; Salminen et al. 2017a). Interestingly, there are many studies indicating that the FGF21 and klotho proteins both possess anti-fibrotic properties in many fibrosis models (Mencke et al. 2017b; Ding et al. 2019; Meng et al. 2021; Wang et al. 2023a, b). For instance, Wang et al. (2023a, b) revealed that FGF21 exposure attenuated the extent of the bleomycin-induced pulmonary fibrosis in mice. Ding et al. (2019) demonstrated that the soluble klotho protein reduced the severity of angiotensin II-induced cardiac fibrosis and hypetrophy in mice by inhibiting the TGF-β signaling pathway. Currently, it is known that klotho and FGF21 proteins can affect several signaling pathways although it seems that the inhibition of TGF-β signaling might be involved in tissue fibrosis (Ding et al. 2019; Wang et al. 2023a, b). There are several investigations indicating that both the klotho and FGF21 proteins are able to activate AMPK signaling in many experimental models (Salminen et al. 2017b; Luo et al. 2023). For instance, Luo et al. (2023) demonstrated that the klotho protein stimulated AMPK signaling which subsequently promoted the phosphorylation and degradation of the YAP protein in mouse kidney. As described above, YAP/TAZ signaling is recognized as an important fibrogenic pathway. In addition, it is known that the FGF21-activated FGF receptor 1 (FGFR1) is able to induce AMPK signaling (Salminen et al. 2017b). Moreover, it has been demonstrated that AMPK signaling activates several down-stream signaling pathways associated with longevity, e.g., it stimulates the FoxO/DAF-16, NRF2/SKN-1, ULK1/autophagy, and SIRT1 pathways, whereas it inhibits the mTOR, NF-κB, and CRTC-1 pathways (Salminen and Kaarniranta 2012). Currently their connections to the myofibroblast differentiation and the age-related fibrosis still need to be clarified. In general, it seems that AMPK signaling is able to inhibit myofibroblast differentiation by suppressing the signaling mechanisms which stimulates the myofibroblast transition although we do not know all molecular mechanisms underpinning the differentiation process, e.g., the role of epigenetic regulation, which control the fate of fibroblasts with aging.

Interestingly, it seems that metabolic processes might also control the differentiation process since it is known that the transition of myofibroblasts is dependent on increased activity of the enzymes involved in aerobic glycolysis (Xie et al. 2015; Ding et al. 2017). For instance, Xie et al. (2015) demonstrated that the expression of key glycolytic enzymes, e.g., 6-phosphofructo-2-kinase/fructose-2,6-biphosphatase 3 (PFKFB3), and the amount of glycolytic flux were up-regulated in the myofibroblasts in mouse fibrotic lungs. They revealed that the inhibition of glycolysis reduced the profibrotic phenotype of myofibroblasts, whereas an increase in aerobic glycolysis promoted the differentiation of myofibroblasts. They also reported that the inhibition of glycolysis attenuated the extent of bleomycin-induced pulmonary fibrosis in mice. Intriguingly, Faubert et al. (2013) demonstrated that AMPK signaling was a potent inhibitor of the Warburg effect in cancer cells, i.e., a phenomenon that increases aerobic glycolysis. Subsequently, Tang et al. (2021) reported that metformin attenuated the LPS-induced collagen synthesis in human lung fibroblasts by inhibiting the activity of PFKFB3 via the AMPK/mTOR pathway. Moreover, Yu et al. (2021) revealed that treatment of mice with 3-bromopyruvate, an inhibitor of aerobic glycolysis, suppressed the TGF-β-induced differentiation of myofibroblasts and subsequently reduced the extent of renal fibrosis. Currently, the reprogramming of glycolysis seems to be a promising therapeutic target to combat fibrotic diseases (Ung et al. 2021).

Interestingly, AMPK signaling itself can be declined during the aging process (Qiang et al. 2007; Reznick et al. 2007; Hardman et al. 2014; Salminen et al. 2016) and this might enhance the age-related myofibroblast differentiation and lead to the accumulation of fibrotic lesions within aged tissues. It seems that it is the responsiveness of AMPK signaling to diverse insults which is impaired with aging rather than the expression of the different subunits of the heterotrimeric AMPK complex. For instance, Reznick et al. (2007) demonstrated that an AICAR infusion into young rats induced a strong activation of the AMPKα2 subunit in EDL muscle, whereas in old mice the exposure to AICAR did not trigger any activation of the AMPKα2 subunit. Aging also impaired the insulin-stimulated glucose uptake into rat skeletal muscle, an effect which was attributed to a decline in the activation of AMPKα (Qiang et al. 2007). Decline in glucose uptake might reduce the level of aerobic glycolysis and disturb the differentiation of myofibroblasts. However, it is not known whether the aging process affects the responsiveness of AMPK signaling in fibroblasts in the microenvironment of aged tissues. Currently, the effect of aging on several inhibitory mechanisms of AMPK signaling, i.e., AMPK phosphatases and inhibitory protein kinases (Salminen et al. 2016), needs to be clarified. Although tissue fibrosis is a well-known age-related phenotype, this does not necessarily mean that an increase in fibrosis could only be attributed to an age-related decline in AMPK signaling. However, it is known that AMPK signaling prevents premature senescence in human fibroblasts and keratinocytes via an inhibition of oxidative stress (Ido et al. 2012; Han et al. 2016). Accordingly, oxidative stress increases with aging as also does cellular senescence, which is an important enhancer of fibrotic lesions, as discussed earlier. In addition, an accumulation of fibrotic material into tissues with aging could be associated with an age-related deficiency of autophagy (Yue et al. 2022) which is recognized to be under the regulation of AMPK signaling (see above). These observations emphasize that an age-related decline in AMPK signaling could accelerate the aging process by enhancing myofibroblast differentiation and deposition of fibrotic lesions.

## Conclusions

Single-cell transcriptome experiments have revealed the remarkable heterogeneity of the tissue fibroblasts involved not only in the aging process but also in many pathological states. Given that fibroblasts are sensors of the tissue environment, they attempt to maintain homeostasis by adapting to the altered microenvironment as well as striving to repair tissue injuries, both in the aging process and pathological states. Fibroblasts are able to display many different phenotypes, such as those with fibrogenic and non-fibrogenic, inflammatory and immunosuppressive, and senescent properties. Interestingly, it is known that several enhancers of the aging process stimulate myofibroblast differentiation both from the tissue-resident fibroblasts and via a transdifferentiation phenomenon from other cell types, especially those of the mesenchymal origin. Myofibroblasts are fibrogenic cells expressing many immunosuppressive properties which have been commonly associated with degenerative processes although they prevent excessive inflammatory responses associated with aging and many age-related diseases. The presence of fibrotic lesions within tissues is a typical hallmark of aging and especially common in age-related diseases. There is abundant evidence that AMPK signaling can suppress the differentiation of fibrogenic myofibroblasts by inhibiting the signaling pathways which are the major inducers of myofibroblast differentiation, such as the TGF-β-induced and the NF-κB-mediated pathways. It is known that metformin, a well-known activator of AMPK signaling, confers many health benefits. Not only can metformin treatment delay the aging process in experimental animals but it has been claimed that this drug can alleviate the symptoms encountered in many age-related diseases, especially those associated with increased fibrotic lesions, such as atherosclerosis, cardiovascular diseases, and idiopathic pulmonary fibrosis. Thus it is not surprising that currently, AMPK signaling is an important drug target for the development of therapies combatting different fibrotic diseases.
